# Differences among a Portuguese cohort of *BRCA* pathogenic/likely pathogenic variants carriers choosing risk-reducing mastectomy or intensive breast surveillance

**DOI:** 10.1007/s00432-023-04663-9

**Published:** 2023-03-27

**Authors:** Sandra Torres, Bárbara Peleteiro, André Magalhães, Luzia Garrido, Susy Costa, José Luís Fougo

**Affiliations:** 1 Faculdade de Medicina da Universidade do Porto, 4200-319 Porto, Portugal; 2 Breast Center, Centro Hospitalar Universitário de São João, 4200-319 Porto, Portugal; 3 EPI Unit, Institute of Public Health, University of Porto, 4050-600 Porto, Portugal; 4 Laboratory for Integrative and Translation Research in Population Health, University of Porto, 4050-600 Porto, Portugal; 5 Medical Genetics Service, Centro Hospitalar e Universitário São João, 4200-319 Porto, Portugal

**Keywords:** BRCA, Breast cancer, Pathogenic/likely pathogenic variants, Risk-reducing options

## Abstract

**Purpose:**

Women with BRCA1 and BRCA2 (BRCA1/2) pathogenic/likely pathogenic (P/LP) variants have a higher risk to develop breast and ovarian cancer. In structured high-risk clinics, risk-reducing measures are adopted. This study aimed at characterizing these women and identify factors that may have influenced their choice between risk reduction mastectomy (RRM) and intensive breast surveillance (IBS).

**Methods:**

This study reviewed retrospectively 187 clinical records of affected and unaffected women with P/LP variants of the BRCA1/2 genes, from 2007 to 2022, of which 50 chose RRM, while 137 chose IBS. The research focused on personal and family history and tumor characteristics and their relation with the preventive option chosen.

**Results:**

Among women with personal history of breast cancer, a higher proportion opted for RRM compared to those asymptomatic (34.2% vs 21.3%, *p* = 0.049), with younger age determining the option for RRM (38.5 years vs 44.0 years, *p* < 0.001). Among women with personal history of ovarian cancer, a higher proportion opted for RRM compared to those without that history (62.5% vs 25.1%, *p* = 0.033), with younger age determining the option for RRM (42.6 years vs 62.7 years, *p* = 0.009). Women who had bilateral salpingo-oophorectomy were more likely to choose RRM than those who did not (37.3% vs 18.3%, *p* = 0.003). Family history was not associated with preventive option (33.3% vs 25.3, *p* = 0.346).

**Conclusions:**

The decision for the preventive option is multifactorial. In our study, personal history of breast or ovarian cancer, younger age at diagnosis, and previous bilateral salpingo-oophorectomy were associated with the choice of RRM. Family history was not associated with the preventive option.

**Supplementary Information:**

The online version contains supplementary material available at 10.1007/s00432-023-04663-9.

## Introduction

Hereditary breast cancer corresponds to 5–10% of all breast cancer cases (Mahdavi et al. 2019). Patients with pathogenic/likely pathogenic (P/LP) variants in a high-penetrance breast cancer susceptibility gene have a significant increased risk of breast cancer. The magnitude is different according to the genes involved. The P/LP variants most frequently associated with early onset hereditary breast cancer are those affecting the *BRCA1* and *BRCA2* (*BRCA1/2)* genes. These variants also increase the risk of developing cancer in other organs, especially in the ovary (Petrucelli et al. 1993).

P/LP variants in *BRCA1*/*2* genes have an autosomal dominant transmission pattern. *BRCAs’* genes are tumor suppressor genes, whose main function is DNA repair homologous recombination (Narod and Foulkes 2004). In patients carrying pathogenic variants, the *BRCA1/2* genes show incomplete penetrance and the cumulative risk at the age of 80 years is up to 72% for breast cancer in *BRCA1* P/LP variants carriers and up to 69% in BRCA2 P/LP variants carriers (Kuchenbaecker et al. 2017).

The increased risk of breast cancer at earlier ages leads to particular concern about preventive strategies in carriers of P/LP variants in *BRCA1/2* genes. These strategies encompass risk reduction mastectomy (RRM) or intensive breast surveillance (IBS). RRM has been shown to reduce breast cancer risk by more than 95% (Ludwig et al. 2016). It is associated with psychological distress decrease, at the cost of persistent body image problems, as it is a major and irreversible procedure (den Heijer et al. 2012). IBS consists of an annual mammogram and breast magnetic resonance imaging (MRI), interspersed, and separated by 6-month intervals and clinical breast examination every half year, starting at the age of 25 (NCCN guidelines for detection, prevention, & risk reduction. Available from: https://www.nccn.org/professionals/physician_gls/#detection).

The aim of this study is to characterize women with P/LP variants in *BRCA1/2* genes who are followed in a High-Risk Breast Clinic, and to identify the factors that influence their choice for one of these breast cancer preventive options: RRM or IBS.

## Patients and methods

Women with known P/LP variants in *BRCA1/2* genes were retrospectively identified at the Breast Center of the Centro Hospitalar Universitário de São João. The cohort consisted of women with *BRCA1/2* P/LP variants diagnosed in the context of the Oncogenetic Clinics of the Centro Hospitalar Universitário de São João (genetic studies were carried out in the Genetic Department) and observed in a High-Risk Breast Clinic at the Breast Center from 30th November 2007 to 12th July 2022, who opted for a breast cancer preventive option: RRM or IBS, according to National Comprehensive Cancer Network guidelines (NCCN guidelines for detection, prevention, and risk reduction. Available from: https://www.nccn.org/professionals/physician_gls/#detection). None of them was treated with chemo-preventive agents.

Clinical information was obtained through the patients’ digital records: age at the first appointment at the High-Risk Breast Clinic; *BRCA1/2* P/LP variants; parity; marital status; education level; smoking status; body mass index (BMI); age at genetic testing; personal history of breast cancer; personal history of ovarian cancer; personal history of other types of cancer; family history of breast cancer; family history of ovarian cancer; and breast cancer preventive option. In case of death, the date and cause of death was recorded.

Women with a personal history of breast cancer were considered symptomatic. In this group of women, more details concerning the breast cancer characteristics were collected: age at diagnosis, histologic type of cancer, type of surgery, other treatments, recurrence, and vital status.

The data collected from the clinical records were analyzed using the IBM^®^ SPSS^®^ Statistics 27 program. Descriptive statistics were used to summarize the main characteristics of the sample. Quantitative variables were summarized by measures of central tendency (mean or median, as applicable) and dispersion (standard deviation) and qualitative variables were summarized by absolute (*N*) and relative (%) frequency. The Chi-square or Fisher’s exact test, as appropriate, was used for comparison of proportions, while Student’s *t* test or Mann–Whitney test was used for comparison of means or medians, respectively. A *p* value < 0.05 was considered statistically significant. Groups of patients who chose IBS or RRM were compared to identify factors related to the preventive option taken.

This study was approved by the Ethics Committee of Centro Hospitalar Universitário de São João and Faculdade de Medicina da Universidade do Porto (CE 124-22). The retrospective nature of the analysis supported the informed consent waiver, for the sake of feasibility. This research ensured the privacy of patient data, since any sort of personal information that allows identification was not used or shown in the database built.

## Results

Overall, 187 carriers of *BRCA1/2* P/LP variants were included, with a median follow-up time of 37 months (0–168 months) from 2007 to 2022.

### Patient characteristics

Table 1 summarizes the characteristics of all patients in the sample. The median age at first high-risk appointment of the women in this sample was 43 (range 18–78), and the median age at the time of genetic testing was 43 (range 18–79). Of the 187 women, 65 (34.8%) were carriers of *BRCA1* P/LP variants and 122 (65.2%) were carriers of *BRCA2* P/LP variants.

**Table 1:** Patient characteristics

	Total [*n* = 187] *N* (%)
Age at first high-risk consultation, years [median (range)]	43.0 (18.0–78.0)
Age at genetic testing, years [median (range)]^a^	43.0 (18.0–79.0)
Altered gene	
*BRCA1*	65 (34.8)
*BRCA2*	122 (65.2)
Parity ^b^	
0	45 (24.9)
≥ 1	136 (75.1)
Marital status^c^	
Married/union of fact	99 (62.7)
Widow/divorced/single	59 (37.3)
Education level^d^	
Elementary school	14 (25.0)
High school	16 (28.6)
University	26 (46.4)
Current smoker^e^	
Non/ex-smoker	97 (80.8)
Current smoker	23 (19.2)
Body Mass Index^f^	
Underweight/normal weight	37 (41.1)
Overweight/obesity	53 (58.9)
Personal history of breast cancer	
No	108 (57.8)
Yes	79 (42.2)
Age at diagnosis, years [median (range)]	42.0 (24.0–81.0)
Personal history of ovarian cancer	
No	179 (95.7)
Yes	8 (4.3)
Age at diagnosis, years [mean (standard deviation)]	50.1 (12.4)
Bilateral salpingo-oophorectomy	
No	104 (55.6)
Yes	83 (44.4)
Personal history of other type of cancer	
No	178 (95.2)
Yes	9 (4.8)
Family history of breast cancer	
No	33 (17.6)
Yes	154 (82.4)
Family history of ovarian cancer	
No	169 (90.4)
Yes	18 (9.6)
Breast cancer preventive option	
Risk-reducing mastectomy	50 (26.7)
Age, years [median (range)]	42.0 (27.0–70.0)
Reconstruction	48 (84.2)
Intensive breast surveillance	137 (73.3)
Deaths	4 (0.02)

^a^Unknown for 7 patients

^b^Unknown for 6 patients

^c^Unknown for 29 patients

^d^Unknown for 56 patients

^e^Unknown for 67 patients

^f^Unknown for 97 patients

Of the 187 women with P/LP variants in *BRCA1/2*, 79 (42.2%) had a personal history of breast cancer with the median age at diagnosis of this cancer of 42 (range 24–81) and 8 (4.3%) had a personal history of ovarian cancer with the mean age at diagnosis of this cancer of 50.1 (standard deviation: 12.4). Bilateral salpingo-oophorectomy was performed in 83 (44.4%) women in this group of carriers, of which 68 (81.9%) were for risk reduction purpose. A total of 9 (4.8%) had personal history of another type of cancer (apart of breast and ovarian), including kidney, pancreas, uterus, lung, brain, and colorectal. Regarding family history, 154 (82.4%) women had a family history of breast cancer, and 18 (9.6%) women had a family history of ovarian cancer.

Following genetic diagnosis, this population of 187 women with *BRCA1/2* P/LP variants was faced with the need to choose the preventive option for breast cancer, with 50 (26.7%) women choosing RRM and 137 (73.3%) women opting for IBS. Of the 50 women who chose RRM, 48 (84.2%) performed breast reconstruction, and of these, only one did not opt for immediate breast reconstruction. Of the women who opted for IBS, six (4.4%) developed breast cancer, with all of them being submitted to RRM of the contralateral breast. Of the asymptomatic women who opted for RRM, two (4%) developed breast cancer later.

Of the entire cohort, four (5%) women died. This corresponds to an overall survival of 95.7% (95% CI 88.3–98.5). Three deaths were not breast cancer related. The other woman died because of breast cancer progression. She belonged to the IBS group. This corresponds to a breast cancer-related mortality of 0.85% (95% CI 0.12–6.07).

### Patient characteristics according to the breast cancer preventive option chosen

Table 2 compares patient characteristics between the two groups: women that opted for RRM and those who selected IBS.

**Table 2:** Patient characteristics according to the breast cancer preventive option chosen

	Risk-reducing mastectomy group [*n* = 50] *N* (%)	Intensive breast surveillance group [*n* = 137] *N* (%)	*p* Value
Age at first high-risk consultation, years [median (range)]	41.0 (28.0–61.0)	44.0 (18.0–78.0)	0.268
Age at genetic testing, years [median (range)]^a^	42.0 (24.0–61.0)	43.0 (18.0–79.0)	0.344
Altered gene			0.051
*BRCA1*	23 (35.4)	42 (64.6)	
*BRCA2*	27 (22.1)	95 (77.9)	
Parity			0.106
0	8 (17.8)	37 (82.2)	
≥ 1	41 (30.1)	95 (69.9)	
Not available/unknown	1	5	
Marital status			0.208
Married/union in fact	31 (31.3)	68 (68.7)	
Widow/divorced/single	13 (22.0)	46 (78.0)	
Not available/unknown	6	23	
Education level			0.737
Elementary school	6 (42.9)	8 (57.1)	
High school	6 (37.5)	10 (62.5)	
University	8 (30.8)	18 (69.2)	
Not available/unknown	33	98	
Smoking status			0.328
Non/ex-smoker	24 (24.7)	73 (75.3)	
Current smoker	8 (34.8)	15 (65.2)	
Not available/unknown	21	46	
Body Mass Index			0.410
Underweight/normal weight	12 (32.4)	25 (67.6)	
Overweight/obesity	13 (24.5)	40 (75.5)	
Not available/unknown	25	72	
Personal history of breast cancer			0.049
No	23 (21.3)	85 (78.7)	
Yes	27 (34.2)	52 (65.8)	
Age at diagnosis, years [median (range)]	38.5 (24.0–56.0)	44.0 (28.0–81.0)	< 0.001
Personal history of ovarian cancer			0.033
No	45 (25.1)	134 (74.9)	
Yes	5 (62.5)	3 (37.5)	
Age at diagnosis, years [mean (standard deviation)]	42.6 (5.4)	62.7 (10.1)	0.009
Bilateral salpingo-oophorectomy			0.003
No	19 (18.3)	85 (81.7)	
Yes	31 (37.3)	52 (62.7)	
Personal history of other type of cancer			0.449
No	47 (26.4)	131 (73.6)	
Yes	3 (33.3)	6 (66.7)	

^a^Unknown for 4 patients in the Risk Reducing Mastectomy Group and 3 patients in the Intensive Breast Surveillance Group

No significant sociodemographic differences were observed between the two groups.

Among those women with personal history of breast cancer, there was a higher proportion who opted for RRM compared to those without personal history of breast cancer (34.2% vs 21.3%, *p* = 0.049). These women were younger at breast cancer diagnosis in the group that chose RRM compared to those who selected IBS (38.5 years old vs 44.0 years oçd, *p* < 0.001).

Likewise, among carriers with personal history of ovarian cancer, there was a higher proportion of women who opted for RRM compared to those without personal history of ovarian cancer (62.5% vs 25.1%, *p* = 0.033). Younger age at diagnosis in women with a history of ovarian cancer also influenced the choice of RRM (42.6 years vs 62.7 years, *p* = 0.009).

Women who had bilateral salpingo-oophorectomy also opted for RRM in a higher proportion compared to those who had not been submitted to bilateral salpingo-oophorectomy (37.3% vs 18.3%, *p* = 0.003).

Among carriers of *BRCA1* P/LP variants, there was a higher proportion that opted for RRM compared to carriers of *BRCA2* P/LP variants (35.4% vs 22.1%, *p* = 0.051), although this was a borderline result.

### Breast cancer characteristics

Table 3 describes the characteristics of breast cancer in women with personal history of breast cancer (*n* = 79). Of the 79 women with a personal history of breast cancer, 52 (65.8%) had P/LP variants in *BRCA2* gene, while 27 (34.2%) had P/LP variants in *BRCA1* gene. Regarding the breast cancer preventive option, 27 (34.2%) opted for RRM, while 52 (65.8%) opted for IBS. Of the 79 breast cancers, 61 (77.2%) were invasive and 18 (22.8%) were ductal in situ. Concerning laterality of the breast cancer, 62 (78.5%) were unilateral and 17 (21.5%) were bilateral.

**Table 3:** Breast cancer characteristics in symptomatic women

	Total [*n* = 79] *N* (%)
Altered gene	
BRCA1	27 (34.2)
BRCA2	52 (65.8)
Breast cancer preventive option	
Risk-reducing mastectomy	27 (34.2)
Intensive breast surveillance	52 (65.8)
Type of cancer	
Invasive breast cancer	61 (77.2)
Ductal carcinoma in situ	18 (22.8)
Laterality of the carcinoma	
Unilateral	62 (78.5)
Bilateral	17 (21.5)
Type of surgery	
Therapeutic mastectomy	52 (65.8)
Reconstruction	36 (64.2)
Breast-conserving surgery	27 (34.2)
Other treatments	
Chemotherapy	67 (84.8)
Radiotherapy	51 (64.6)
Hormone therapy	49 (62.0)
Recurrence	7 (8.9)

All these symptomatic women underwent surgery, with 52 (65.8%) having therapeutic mastectomy and 27 (34.2%) having breast-conserving surgery. Of the 52 women who underwent therapeutic mastectomy, 36 (69.2%) had reconstruction, and of these, 25 (69.4%) were immediate reconstructions. In the group who underwent mastectomy as treatment for their breast cancer (*n* = 52), three mastectomies had an initially risk reduction intention, considering that only in the final pathology result of the surgical specimen, the cancer diagnosis was revealed. In addition to surgical treatment, 67 (84.8%) of the symptomatic women performed chemotherapy, 51 (64.6%) radiotherapy, and 49 (62.0%) hormone therapy.

Of these 79 women with a personal history of breast cancer, seven (8.9%) had disease recurrence, three of them with local recurrence, one with regional recurrence, and three with distant recurrence (one bone, one lung, and one pelvic). Of these seven patients, five chose RRM after the initial diagnosis of breast cancer, therefore before recurrence, and two opted for RRM only after recurrence.

### Family history

Table 4 compares family history according to the breast cancer preventive option chosen.

**Table 4:** Family history according to the breast cancer preventive option

	Risk-reducing mastectomy group [*n* = 57] *N* (%)	Intensive breast surveillance group [*n* = 130] *N* (%)	*p* Value
Family history of breast cancer			0.346
No	11 (33.3)	22 (66.7)	
Yes	39 (25.3)	115 (74.7)	
Number of relatives with breast cancer			0.243
1	9 (19.1)	38 (80.9)	
> 1	30 (28.0)	77 (72.0)	
Age of the relatives at breast cancer diagnosis^a^			0.810
< 40 years	17 (25.8)	49 (74.2)	
≥ 40 years	18 (24.0)	57 (76.0)	
First-degree relatives with breast cancer			0.436
No	21 (30.0)	49 (70.0)	
Yes	29 (24.8)	88 (75.2)	
Number of first-degree relatives with breast cancer			0.710
1	17 (23.6)	55 (76.4)	
> 1	12 (26.7)	33 (73.3)	
Second-degree relatives with breast cancer			0.809
No	28 (27.5)	74 (72.5)	
Yes	22 (25.9)	63 (74.1)	
Number of second-degree relatives with breast cancer			0.732
1	12 (24.5)	37 (75.5)	
> 1	10 (27.8)	26 (72.2)	
Third-degree relatives with breast cancer			0.326
No	34 (24.8)	103 (75.2)	
Yes	16 (32.0)	34 (68.0)	
Number of third-degree relatives with breast cancer			0.697
1	8 (29.6)	19 (70.4)	
> 1	8 (34.8)	15 (65.2)	
Male family members with breast cancer			0.008
No	50 (29.1)	122 (70.9)	
Yes	0 (0.0)	15 (100.0)	
Number of male family members with breast cancer			-
1	0 (0.0)	11 (100.0)	
> 1	0 (0.0)	4 (100.0)	
Family history of deaths from breast cancer			0.106
No	40 (30.1)	93 (69.9)	
Yes	10 (18.5)	44 (81.5)	
Number of deaths from breast cancer			0.549
1	8 (19.5)	33 (80.5)	
> 1	2 (15.4)	11 (84.6)	
Age of the relatives at death from breast cancer^b^			0.461
< 40 years	4 (23.5)	13 (76.5)	
≥ 40 years	6 (18.2)	27 (81.8)	
Family history of ovarian cancer			0.555
No	45 (26.6)	124 (73.4)	
Yes	5 (27.8)	13 (72.2)	
Number of relatives with ovarian cancer			0.225
1	4 (40.0)	6 (60.0)	
> 1	1 (12.5)	7 (87.5)	
Age of the relatives at ovarian cancer diagnosis^c^			0.667
< 40 years	0 (0.0)	1 (100.0)	
≥ 40 years	4 (36.4)	7 (63.6)	
First-degree relatives with ovarian cancer			0.603
No	47 (26.7)	129 (73.3)	
Yes	3 (27.3)	8 (72.7)	
Number of first-degree relatives with ovarian cancer			0.727
1	3 (30.0)	7 (70.0)	
> 1	0 (0.0)	1 (100.0)	
Second-degree relatives with ovarian cancer			0.529
No	47 (26.6)	130 (73.4)	
Yes	3 (30.0)	7 (70.0)	
Number of second-degree relatives with ovarian cancer			0.033
1	3 (75.0)	1 (25.0)	
> 1	0 (0.0)	6 (100.0)	

^a^Unknown for 4 patients in the Risk Reducing Mastectomy Group and 9 patients in the Intensive Breast Surveillance Group

^b^Unknown for 4 patients in the Intensive Breast Surveillance Group

^c^Unknown for 1 patient in the Risk Reducing Mastectomy Group and 5 patients in the Intensive Breast Surveillance Group

All of those who had male family members with breast cancer chose IBS (*p* = 0.008). While all carriers with more than one second-degree relative with ovarian cancer also opted for IBS, only 25% of those with only one second-degree relative with ovarian cancer chose the same breast cancer preventive approach (*p* = 0.033).

There were no significant differences related neither to family history of breast cancer nor ovarian cancer.

## Discussion

This study reviewed retrospectively women with these P/LP variants in *BRCA1/2* followed in a High-Risk Breast Clinic, including patients with breast cancer, with the aim to characterize this population and identify whether there are clinical, demographic, or family characteristics that could be determinant when choosing the preventive strategy.

Previous studies have shown that decision-making regarding options for coping with increased risk of breast cancer is strongly influenced by whether women know they are carriers of P/LP variants in *BRCA1/2* or not*.* It has been clear that these women prefer more aggressive treatments, including risk-reducing surgeries compared to women without P/LP variants in *BRCA1/2* (Stolier and Corsetti 2005). In our patient population, consisting only of *BRCA1/2* P/LP variants carriers, 26.7% underwent RRM, compared to the literature that shows percentages that can reach 50% (Metcalfe et al. 2019). However, this number is expected to grow in the future, because data illustrate that rates of RRM have been rising over the past 20 years (Jung et al. 2020). In our sample, a more marked increase has been observed especially in more recent years, with nearly 1/4 being performed before 2015 and the other 3/4 since then.

In this study, among carriers of P/LP variants in *BRCA1*, there was a higher proportion who opted for RRM compared to carriers of P/LP variants in *BRCA2*, in line with the previous findings (Gilbert et al. 2017), although it was a borderline result. P/LP variants in *BRCA1* confer a higher risk for breast cancer compared to P/LP variants in *BRCA2* (Saslow et al. 2007) and RRM in women with asymptomatic *BRCA1* P/LP variants has been shown to have improved survival compared to those who opted for IBS (Heemskerk-Gerritsen et al. 2019).

Women in our study population opted for RRM more often than IBS when they had personal history of breast cancer. The same occurred to women who had personal history of ovarian cancer. Also, the majority and the most recent studies corroborate our finding (Gilbert et al. 2017; Wei et al. 2021). Only about one-third of women submitted to chemotherapy opted for RRM. Due to the morbidity associated with chemotherapy, we expected that more women who underwent this treatment would opt for RRM as a preventive option for contralateral breast cancer.

Bilateral salpingo-oophorectomy not only reduces the risk of ovarian cancer of up to 80%, but also decreases the risk of developing breast cancer, especially in women before menopause who carry P/LP variants in *BRCA 1/2* (Eisen et al. 2005). The literature showed that the combination of bilateral salpingo-oophorectomy and RRM provides a significant survival benefit (Ingham et al. 2013). We also found that a higher proportion of women with a bilateral salpingo-oophorectomy opted for RRM when compared to those who did not, probably justified by the predisposition of women to undergo risk reduction surgeries (Gilbert et al. 2017).

Of all the factors studied, the presence of males with breast cancer in the family and more than one second-degree relative with ovarian cancer were associated with the choice for undergoing IBS. Although these were statistically significant results, the analysis was based on a very low number of cases.

A stronger family history confers a higher lifetime risk to develop breast cancer among carriers of P/LP variants in *BRCA 1/2* (Petrucelli et al. 1993; Kuchenbaecker et al. 2017), and this can impact decision-making regarding preventive choice. In addition, seeing family members undergoing cancer treatment or dying from breast cancer may also impact emotional decision-making (Henry et al. 2019). In this study, family history up to third-degree in breast cancer and second-degree in ovarian cancer did not have the expected impact in this population regarding the choice for the preventive option, as described. These results suggested that family history did not play an important role in self-risk perception in these women.

In this study, there were no significant differences in the age of genetic testing between the two groups, although younger women opted for RRM. These women were only proposed for RRM after the age of 40 years, as this is the age after which a significant increase in the development of breast cancer has been demonstrated, and normally, the reproductive project is completed (Mavaddat et al. 2013). Some studies showed that younger patients tend to choose IBS because of their desire to keep their breasts, for breastfeeding or to keep the body appearance, or because providers may not recommend RRM to young population, due to improvement in the early breast cancer detection capacity of MRI screening and in the overall risk reduction of IBS (Daly et al. 2021). Others have shown that younger age is a positive predictor for performing RRM (Manoukian et al. 2019).

Some trials concluded that women in the RRM group were more likely to be married or living with someone and having children compared to those in the surveillance group. This was also showed by us and, although the results were not statistically significant, this could be clinically relevant. The fear of leaving young children behind and the desire to live longer for the family is a significant factor in decision to undergo RRM (Haroun et al. 2011).

Women with a personal history of breast cancer accounted for almost half of the study population. There was no difference regarding bilateral carcinoma between BRCA1 and BRCA2 carriers (22.2% vs 23.1%, respectively). This was not predictable considering some studies that showed a 15-year actuarial risk of contralateral breast cancer of 36.1% for women with P/LP variants *BRCA1* and 28.5% for women with P/LP variants in *BRCA2* (Metcalfe et al. 2011). Another reason why women with P/LP variants in *BRCA1/2* tend to undergo therapeutic mastectomy in greater proportion compared to women who do not carry these pathogenic variants is because of the higher risk of local failure to control the disease (King et al. 2015). In this symptomatic women population, 65.8% performed therapeutic mastectomy. Breast cancer recurrence in our population (8.9%) was higher than in the sporadic population undergoing mastectomy (2–3% local recurrence, 2–3% regional recurrence, and 7–9% distant recurrence) (Bargon et al. 2022). An important aspect is that these seven patients had chosen RRM after the initial breast cancer diagnosis, and five out of the seven made their choice before the recurrence, so in these women, recurrence did not seem to be an influential factor in choosing preventive surgery.

To the best of our knowledge, this is the first characterization of women with P/LP variants in *BRCA1/2* in Portugal, regarding the choice for the preferred breast cancer preventive option.

These women were diagnosed, followed, and treated at a certified center, annually audited with several quality indicators (Biganzoli et al. 2017, 2020). Another strength of this study is the long follow-up (with a median of 37 months) of the patients in this high-risk clinic. Besides, it is important to emphasize some of the patients are followed since 2007.

The guidelines for the risk management of women with P/LP variants in *BRCA1/2* have remained stable since the beginning of the follow-up period for the patients in this study.

However, the study has some limitations. This study is retrospective in nature. The guidelines for genetic testing and integration into the High-Risk Breast Clinic have changed over the patients' follow-up time, so it is possible that some patients who would currently make sense to be part of the study population may have been missed. The fact that this study was carried out with a population from only one institution may potentially limit the generalizability to other populations, from different geographic locations, with different cultures and/or with limited access to specialized health care. Another limitation of this study was the incomplete recording of several of the variables investigated as possible determinants of the decision-making process.

## Conclusions

The choice of RRM over IBS was significantly associated with the existence of personal history of breast cancer, personal history of ovarian cancer, previous bilateral salpingo-oophorectomy, younger age at breast cancer diagnosis, and younger age at ovarian cancer diagnosis.

Family history of breast cancer and family history of ovarian cancer did not have the impact that we had expected and described by most of the literature, regarding the choice for the breast cancer preventive option. Therefore, contrary to what was predicted, family history did not play an important role in these women's perceived risk.

The data from this study highlight factors that may be important in women carrying P/LP variants in *BRCA1/2* and that may help to advise these women when they are faced with this decision.

## Supplementary Information

Below is the link to the electronic supplementary material.

Supplementary file1 (DOCX 30 KB)

## Data Availability

Data are not freely available as genetic data are sensitive data, for which we are obliged legally and ethically in our country to restrict the access due to preserving patient privacy.
