# Supplementary material for: Differences among a Portuguese cohort of BRCA pathogenic/likely pathogenic variants carriers choosing risk-reducing mastectomy or intensive breast surveillance
Source: J Cancer Res Clin Oncol. 2023 Mar 27;149(10):7529–38. doi: 10.1007/s00432-023-04663-9 (PMC10374730; doi:10.1007/s00432-023-04663-9)
Supplement: Supplementary file 1 — Supplementary file1 (DOCX 30 KB) [file 432_2023_4663_MOESM1_ESM.docx]

**TABLES**

**Table 1.** Patient Characteristics

|  | Total [n=187]  N (%) |
| --- | --- |
| Age at first high-risk consultation, years [median (range)] | 43.0 (18.0-78.0) |
| Age at genetic testing, years [median (range)] ^a^ | 43.0 (18.0-79.0) |
| Altered gene |  |
| *BRCA1* | 65 (34.8) |
| *BRCA2* | 122 (65.2) |
| Parity |  |
| 0 | 45 (24.9) |
| ≥1 | 136 (75.1) |
| *Not available/unknow* | *6* |
| Marital status |  |
| Married/Union of fact | 99 (62.7) |
| Widow/Divorced/Single | 59 (37.3) |
| *Not available/unknow* | *29* |
| Education Level |  |
| Elementary school | 14 (25.0) |
| High school | 16 (28.6) |
| University | 26 (46.4) |
| *Not available/unknow* | *56* |
| Current smoker |  |
| Non/Ex-smoker | 97 (80.8) |
| Current smoker | 23 (19.2) |
| *Not available/unknow* | *67* |
| Body Mass Index |  |
| Underweight/Normal weight | 37 (41.1) |
| Overweight/Obesity | 53 (58.9) |
| *Not available/unknow* | *97* |
| Personal history of breast cancer |  |
| No | 108 (57.8) |
| Yes | 79 (42.2) |
| Age at diagnosis, years [median (range)] | 42.0 (24.0-81.0) |
| Personal history of ovarian cancer |  |
| No | 179 (95.7) |
| Yes | 8 (4.3) |
| Age at diagnosis, years [mean (standard deviation)] | 50.1 (12.4) |
| Bilateral salpingoophorectomy |  |
| No | 104 (55.6) |
| Yes | 83 (44.4) |
| Personal history of other type of cancer |  |
| No | 178 (95.2) |
| Yes | 9 (4.8) |
| Family history of breast cancer |  |
| No | 33 (17.6) |
| Yes | 154 (82.4) |
| Family history of ovarian cancer |  |
| No | 169 (90.4) |
| Yes | 18 (9.6) |
| Breast cancer preventive option |  |
| Risk reducing mastectomy | 50 (26.7) |
| Age, years [median (range)] | 42.0 (27.0-70.0) |
| Reconstruction | 48 (84.2) |
| Intensive breast surveillance | 137 (73.3) |
| Deaths | 4 (0.02) |

^a^ Not available/unknown for 7 patients.

**Table 2.** Patient Characteristics According to the Breast Cancer Preventive Option Chosen

|  | Risk Reducing Mastectomy Group [*n*=50]  N (%) | Intensive Breast Surveillance Group [*n*=137]  N (%) | p value |
| --- | --- | --- | --- |
| Age at first high-risk consultation, years [median (range)] | 41.0 (28.0-61.0) | 44.0 (18.0-78.0) | 0.268 |
| Age at genetic testing, years [median (range)] ^a^ | 42.0 (24.0-61.0) | 43.0 (18.0-79.0) | 0.344 |
| Altered gene |  |  | 0.051 |
| *BRCA1* | 23 (35.4) | 42 (64.6) |  |
| *BRCA2* | 27 (22.1) | 95 (77.9) |  |
| Parity |  |  | 0.106 |
| 0 | 8 (17.8) | 37 (82.2) |  |
| ≥1 | 41 (30.1) | 95 (69.9) |  |
| *Not available/unknow* | *1* | *5* |  |
| Marital status |  |  | 0.208 |
| Married/Union in fact | 31 (31.3) | 68 (68.7) |  |
| Widow/Divorced/Single | 13 (22.0) | 46 (78.0) |  |
| *Not available/unknow* | *6* | *23* |  |
| Education Level |  |  | 0.737 |
| Elementary school | 6 (42.9) | 8 (57.1) |  |
| High school | 6 (37.5) | 10 (62.5) |  |
| University | 8 (30.8) | 18 (69.2) |  |
| *Not available/unknow* | *33* | *98* |  |
| Smoking status |  |  | 0.328 |
| Non/Ex-smoker | 24 (24.7) | 73 (75.3) |  |
| Current smoker | 8 (34.8) | 15 (65.2) |  |
| *Not available/unknow* | *21* | *46* |  |
| Body Mass Index |  |  | 0.410 |
| Underweight/Normal weight | 12 (32.4) | 25 (67.6) |  |
| Overweight/Obesity | 13 (24.5) | 40 (75.5) |  |
| *Not available/unknow* | *25* | *72* |  |
| Personal history of breast cancer |  |  | 0.049 |
| No | 23 (21.3) | 85 (78.7) |  |
| Yes | 27 (34.2) | 52 (65.8) |  |
| Age at diagnosis, years [median (range)] | 38.5 (24.0-56.0) | 44.0 (28.0-81.0) | < 0.001 |
| Personal history of ovarian cancer |  |  | 0.033 |
| No | 45 (25.1) | 134 (74.9) |  |
| Yes | 5 (62.5) | 3 (37.5) |  |
| Age at diagnosis, years [mean (standard deviation)] | 42.6 (5.4) | 62.7 (10.1) | 0.009 |
| Bilateral salpingoophorectomy |  |  | 0.003 |
| No | 19 (18.3) | 85 (81.7) |  |
| Yes | 31 (37.3) | 52 (62.7) |  |
| Personal history of other type of cancer |  |  | 0.449 |
| No | 47 (26.4) | 131 (73.6) |  |
| Yes | 3 (33.3) | 6 (66.7) |  |

^a^ Not available/unknown for 4 patients in the Risk Reducing Mastectomy Group and 3 patients in the Intensive Breast Surveillance Group.

**Table 3.** Breast Cancer Characteristics in Symptomatic Women

|  | Total [n=79]  N (%) |
| --- | --- |
| Altered gene |  |
| BRCA1 | 27 (34.2) |
| BRCA2 | 52 (65.8) |
| Breast cancer preventive option |  |
| Risk reducing mastectomy | 27 (34.2) |
| Intensive breast surveillance | 52 (65.8) |
| Type of cancer |  |
| Invasive breast cancer | 61 (77.2) |
| Ductal carcinoma in situ | 18 (22.8) |
| Laterality of the Carcinoma |  |
| Unilateral | 62 (78.5) |
| Bilateral | 17 (21.5) |
| Type of surgery |  |
| Therapeutic mastectomy | 52 (65.8) |
| Reconstruction | 36 (64.2) |
| Breast-conserving surgery | 27 (34.2) |
| Other treatments |  |
| Chemotherapy | 67 (84.8) |
| Radiotherapy | 51 (64.6) |
| Hormone therapy | 49 (62.0) |
| Recurrence | 7 (8.9) |
|  |  |

**Table 4.** Family History According to the Breast Cancer Preventive Option

|  | Risk Reducing Mastectomy Group [*n*=57]  N (%) | Intensive Breast Surveillance Group [*n*=130]  N (%) | p value |
| --- | --- | --- | --- |
| **FAMILY HISTORY OF BREAST CANCER** |  |  | 0.346 |
| No | 11 (33.3) | 22 (66.7) |  |
| Yes | 39 (25.3) | 115 (74.7) |  |
| **Number of relatives with breast cancer** |  |  | 0.243 |
| 1 | 9 (19.1) | 38 (80.9) |  |
| >1 | 30 (28.0) | 77 (72.0) |  |
| **Age of the relatives at breast cancer diagnosis** |  |  | 0.810 |
| < 40 years | 17 (25.8) | 49 (74.2) |  |
| ≥ 40 years | 18 (24.0) | 57 (76.0) |  |
| *Not available/unknown* | *4* | *9* |  |
| **First-degree relatives with breast cancer** |  |  | 0.436 |
| No | 21 (30.0) | 49 (70.0) |  |
| Yes | 29 (24.8) | 88 (75.2) |  |
| **Number of first-degree relatives with breast cancer** |  |  | 0.710 |
| 1 | 17 (23.6) | 55 (76.4) |  |
| >1 | 12 (26.7) | 33 (73.3) |  |
| **Second-degree relatives with breast cancer** |  |  | 0.809 |
| No | 28 (27.5) | 74 (72.5) |  |
| Yes | 22 (25.9) | 63 (74.1) |  |
| **Number of second-degree relatives with breast cancer** |  |  | 0.732 |
| 1 | 12 (24.5) | 37 (75.5) |  |
| >1 | 10 (27.8) | 26 (72.2) |  |
| **Third-degree relatives with breast cancer** |  |  | 0.326 |
| No | 34 (24.8) | 103 (75.2) |  |
| Yes | 16 (32.0) | 34 (68.0) |  |
| **Number of third-degree relatives with breast cancer** |  |  | 0.697 |
| 1 | 8 (29.6) | 19 (70.4) |  |
| >1 | 8 (34.8) | 15 (65.2) |  |
| **Male family members with breast cancer** |  |  | 0.008 |
| No | 50 (29.1) | 122 (70.9) |  |
| Yes | 0 (0.0) | 15 (100.0) |  |
| **Number of male family members with breast cancer** |  |  | - |
| 1 | 0 (0.0) | 11 (100.0) |  |
| >1 | 0 (0.0) | 4 (100.0) |  |
| **Family history of deaths from breast cancer** |  |  | 0.106 |
| No | 40 (30.1) | 93 (69.9) |  |
| Yes | 10 (18.5) | 44 (81.5) |  |
| **Number of deaths from breast cancer** |  |  | 0.549 |
| 1 | 8 (19.5) | 33 (80.5) |  |
| >1 | 2 (15.4) | 11 (84.6) |  |
| **Age of the relatives at death from breast cancer** |  |  | 0.461 |
| < 40 years | 4 (23.5) | 13 (76.5) |  |
| ≥ 40 years | 6 (18.2) | 27 (81.8) |  |
| *Not available/unknown* |  | *4* |  |
| **FAMILY HISTORY OF OVARIAN CANCER** |  |  | 0.555 |
| No | 45 (26.6) | 124 (73.4) |  |
| Yes | 5 (27.8) | 13 (72.2) |  |
| **Number of relatives with ovarian cancer** |  |  | 0.225 |
| 1 | 4 (40.0) | 6 (60.0) |  |
| >1 | 1 (12.5) | 7 (87.5) |  |
| **Age of the relatives at ovarian cancer diagnosis** |  |  | 0.667 |
| < 40 years | 0 (0.0) | 1 (100.0) |  |
| ≥ 40 years | 4 (36.4) | 7 (63.6) |  |
| *Not available/unknown* | *1* | *5* |  |
| **First-degree relatives with ovarian cancer** |  |  | 0.603 |
| No | 47 (26.7) | 129 (73.3) |  |
| Yes | 3 (27.3) | 8 (72.7) |  |
| **Number of first-degree relatives with ovarian cancer** |  |  | 0.727 |
| 1 | 3 (30.0) | 7 (70.0) |  |
| >1 | 0 (0.0) | 1 (100.0) |  |
| **Second-degree relatives with ovarian cancer** |  |  | 0.529 |
| No | 47 (26.6) | 130 (73.4) |  |
| Yes | 3 (30.0) | 7 (70.0) |  |
| **Number of second-degree relatives with ovarian cancer** |  |  | 0.033 |
| 1 | 3 (75.0) | 1 (25.0) |  |
| >1 | 0 (0.0) | 6 (100.0) |  |
